# Ultrasound-guided paravertebral blockade reduced perioperative opioids requirement in pancreatic resection: A randomized controlled trial

**DOI:** 10.3389/fsurg.2022.903441

**Published:** 2022-08-30

**Authors:** Ye Han, Yuanqiang Dai, Yaping Shi, Xiaoxiu Zhang, Boyang Xia, Qiufang Ji, Xiya Yu, Jinjun Bian, Tao Xu

**Affiliations:** Faculty of Anesthesiology, Changhai Hospital, Naval Medical University, Shanghai, China

**Keywords:** paravertebral blockade, surgery, IL-10, opioids dosage, RCT

## Abstract

**Background:**

Perioperative opioid use for pain control has been found to be associated with side effects and adverse prognosis. In this study, we hypothesized that paravertebral block could reduce the consumption of opioids during pancreatic resection surgery.

**Methods:**

We conducted a prospective, randomized trial. Patients with resectable pancreatic cancer were randomly assigned to one of the two groups: those who received bilateral paravertebral block combined with general anesthesia [bilateral paravertebral blockade (PTB) group] or those who received only general anesthesia (Control group). The primary endpoint was the perioperative consumption of opioids (sufentanil and remifentanil). The main secondary endpoints were pain scores, complications, and serum cytokine levels.

**Results:**

A total of 153 patients were enrolled in the study and 119 cases were analyzed. Compared to the control group, patients in PTB patients had significantly lower perioperative (30.81 vs. 56.17 µg), and intraoperative (9.58 vs. 33.67 µg) doses of sufentanil (both *p* < 0.001). Numerical rating scale scores of pain were comparable between the two groups. No statistical differences in complications were detected.

**Conclusion:**

Bilateral paravertebral block combined with general anesthesia reduced the perioperative consumption of opioids by 45%.

**Registration number:**

ChiCTR1800020291 (available on http://www.chictr.org.cn/).

## Introduction

Surgical resection provides the only possible cure for pancreatic cancer (1, 2). Opioids such as fentanyl and its derivatives (sufentanil and remifentanil), and morphine, are considered as the pillar stones of pain management during the surgical process (3). However, opioid usage has been reported to be associated with both acute and chronic undesirable side effects (4, 5). In addition, it may result in higher risks of cancer disease progression due to the functions through various receptors (6–8). Thus, reduction of opioids, a common goal in surgical analgesia strategy, may be even more important in cancer patients undergoing pancreatic resections.

Optimizing perioperative pain management is of great importance for limiting opioid usage following surgery, both in the hospital and after discharge. It has been suggested that acute postoperative pain is closely associated with chronic pain development. For example, acute pain during the first 9 h after breast cancer surgery is a critical predictor of chronic pain and long-term analgesic requirements (9). Moreover, the severity of acute postpartum pain was an independent risk factor for persistent pain and depression (10). In addition, certain opioid-induced adverse events, such as respiratory depression, mostly occur within the first 24 h after surgery (11, 12). Therefore, acute pain management and opioid usage during the immediate period after surgery need to be carefully managed.

Regional anesthesia techniques provide an important synergistic method of pain control in abdominal surgeries (13). Thoracic epidural block has been suggested to be able to provide excellent analgesia and reduce perioperative opioid consumption (14), as well as improve oncological outcomes and survival (15). However, only a small number of retrospective studies in pancreatic resections have been reported (15). In addition, an epidural block may be technically challenging (16), with potential contradiction in elderly and underweight patients (17, 18), as well as side effects such as hypotension (19, 20).

A paravertebral block is a regional anesthetic technique where local anesthetics are injected into the paravertebral space to induce ipsilateral somatic and sympathetic nerve block (21). The technique had well-documented benefits comparable to epidural anesthesia (22, 23). However, very few clinical studies have been performed on its effects in the pancreatic section (24).

We performed a randomized controlled study to test the hypothesis that paravertebral block could reduce the consumption of opioids in pancreatic resection surgery.

## Materials and methods

### Ethics approval and clinical registration

The study was conducted in accordance with the ethical standard specified by the National Health Commission of the People's Republic of China (Act 11, 2016) and was approved by the ethics committee of Changhai Hospital (CHEC2020-015). All patients presented their written informed consent before enrollment. The clinical registration number is ChiCTR1800020291 (available on http://www.chictr.org.cn/).

### Study design

This was a prospective, randomized, controlled interventional study, in which patients were randomly assigned at a ratio of 1:1 into two groups: either those who received bilateral paravertebral block in addition to general anesthesia (PTB group) or those who received sham procedure (taping catheters on the skin surface at the same sites as paravertebral block) in addition to general anesthesia (control group).

### Patients

From 1 May 2019 to 30 November 2019, patients hospitalized in the Third General Surgery Department of Changhai Hospital, Shanghai, China with a diagnosis of pancreatic cancer were recruited. The inclusion criteria were the following: (1) age between 18 and 70 years; (2) undergoing open radical resection of pancreatic cancer; (3) American Society of Anesthesia (ASA) I–II; and (4) signed informed consent. The exclusion criteria are the following: (1) preoperative radiotherapy or chemotherapy; (2) history of chronic pain, use of analgesics preoperatively; (3) hormone or steroids treatment before surgery; (4) previous neuromuscular system disease; (5) previous cognitive disorders; (6) contradictions to paravertebral block (including severe coagulation disorders, infections or allergies to local anesthetics); (7) body mass index (BMI) ≥ 30 kg/m^2^, (8) severe organic heart disease or arrhythmia; and (9) receiving palliative surgery, emergency surgery or reoperation.

### Interventions

In the PTB group, an ultrasound-guided bilateral paravertebral blockade was performed by a senior anesthesiologist. With the patient lying in a prone position, the block was performed with a CONTIPLEX™ FX nerve block tray (BBraun Melsungen AG, 34209 Melsungen, Germany). The imaginary horizontal line connecting T7 to the lower tip of the scapula was used as a marker for recognition of T7 or T8. Local infiltration analgesic was performed with topical application of 1% lidocaine (1–3 ml) on the lateral site of the spinous process. The needle was inserted with an in-plane approach, passing through the costotransverse ligament into the paravertebral space under ultrasound guidance. Correct placement of the needle was confirmed by the injection of a small amount of saline. Infiltration of 20 ml of 0.375% ropivacaine and epinephrine (1:200,000) was performed if there was no blood, cerebrospinal fluid, or gas in aspiration after the appearance of anterior displacement of pleura. Then the catheter was pushed forward 1–2 mm to surpass the needle tip, and the needle was withdrawn carefully. The above procedures were repeated on both sides. After the completion of the block for 15–20 min, the sensory block level (indicated by the loss of acupuncture sensation at 2–3 or more ipsilateral vertebral dermatome) was tested and anesthesia was induced. Another 20 ml of 0.375% ropivacaine was injected into the paravertebral space *via* the catheters bilaterally 1 h before the surgery is over and the catheters were removed subsequently. If continuous administration of vasoactive drugs was warranted to maintain blood pressure during operation, the amount of ropivacaine was reduced by half, that is, 10 ml for each lateral.

In the control group, the catheters were taped at the same positions as the PTB group on the skin surface before induction. The same dosage of local anesthetics was administered through the catheters 1 h before the end of the operation by another anesthesiologist who took over after induction without knowing the intervention method.

Patients in both groups received general anesthesia. For induction, dexmedetomidine 1 µg/kg was initially administered followed by etomidate (0.2–0.3 µg/kg), sufentanil (0.2–0.4 µg/kg), cisatracurium (0.04 mg/kg), midazolam (0.05–0.1 mg/kg), and dexamethasone (8 mg) 10 min later. Tracheal intubation and mechanical ventilation were then carried out. The settings for mechanical ventilation were listed as follows: tidal volume at 6–8 ml/kg, respiratory rate at 10–12 times/min, inspiratory-to-expiratory ratio at 1:2, and partial pressure for end-tidal carbon dioxide at 35–45 mmHg. Radial artery catheterization and jugular vein catheterization were performed after anesthetic induction. Maintenance of anesthesia was achieved by inhalation of sevoflurane 1–1.5 minimum alveolar concentration (MAC) and intermittent intravenous bolus of cisatracurium. Cerebral state index (CSI) was monitored by bispectral index (BIS) and maintained within the range of 40–60. A bolus of 5–10 µg sufentanil was supplemented if mean arterial pressure (MAP) or heart rate (HR) was 20% greater than the baseline value. Hypotension (reduction in blood pressure of more than 20%) was treated i.v. with an expedited infusion of phenylephrine (50 µg) or ephedrine (6 mg). Atropine (0.3–0.5 mg) was given if bradycardia (HR < 60 bpm) occurred. Ephedrine (6 mg) was administered if the heart rate was less than 50 bpm and the MAP was less than 65 mmHg. Patient-controlled intravenous analgesia (PCIA) pump was programmed to rescue analgesia, delivering a solution of butorphanol (4 mg), sufentanil (1.5 µg/kg), and flurbiprofen (5 mg/kg) diluted in 100 ml with saline, with loading dose at 2 ml and background dose at 2 ml/h. The single amount of PCIA was 2 ml and a lockout interval was set at 10 min. The patients were transferred to the intensive care unit (ICU) after surgery, and tracheal tubes were removed after the patient became sober. The numerical rating scale (NRS) was assessed and recorded by nursing staff in the ICU at 6 and 12 h postoperatively. Within 24 h, for patients with NRS > 3, remifentanil was applied *via* intravenous bolus pump (dosage at 0.01–0.05 µg/kg/min) until the score was less than 3 (25). Parecoxib (dosage at 40 mg) was administered for analgesic rescue, 24 h after surgery.

### Assessment of outcomes

#### Primary outcome

The primary outcome was the perioperative consumption of opioids (measured in sufentanil and remifentanil), which was defined as the total dosage of sufentanil administrated from before to 24 h after surgery.

#### Secondary outcomes

Secondary outcomes measured postoperative levels of serum inflammatory markers, including IL-6, IL-8, IL-10, IL-1β, IL-2R, TNF-α, C-reactive protein (CRP), and Treg. Other secondary measures included subjective assessment of pain, opioid side effects, possible surgical and paravertebral block complications, consumption of vasoactive drug, intraoperative fluid volume, postoperative exhaust time, and total length of stay.

The levels of cytokines were detected at 6 h in the postoperative period, including IL-6, IL-8, IL-10, IL-1β, IL-2R, and TNF-α. The counts of Treg at 24 h after the surgery were measured by flow cytometry (26). Postoperative pain was assessed using an 11-point NRS at 12 and 24 h after surgery. Adverse effects associated with opioids were assessed by a simplified postoperative nausea and vomiting impact scale (SPONVIS), which is a 4-point numerical scale (0 = no PONV, 1 = mild nausea, 2 = severe nausea or vomiting once, and 3 = vomiting more than once) at 24 h after surgery. Surgical complications including infection, bleeding, anastomotic leak, stress ulcer, and cardiocerebrovascular events were collected from medical records. Relevant complications of paravertebral block such as infection, local hematoma, and pneumothorax were retrieved from anesthetic records. Consumption of vasoactive drugs and intraoperative fluid volume were recorded during the operation. Anal exhaust time and total length of stay were provided by doctors responsible for follow-up.

### Sample size

Sample size estimation was based on the following principles. The bilateral paravertebral block could reduce the amount of sufentanil by 20% perioperatively and the average dose of sufentanil was 26.4 ± 9.5 µg in the pilot study. To have an 80% power, with type I error (*α*) of 0.05, 53 cases were needed for each arm based on the calculation in the PASS software. Given the rate of withdrawal and loss to follow-up was 20%, 128 cases in total were required for the sample size.

### Randomization and blinding

All patients were randomly assigned to the PTB group and the control group according to a pre-set randomized number table. The allocation sequence was concealed to patients and anesthetists in the operation room, but not for the senior anesthesiologist who performed the paravertebral block or taped the catheters. The anesthesiologist in the operation room, nurses in the ICU, and doctors responsible for looking after the patients during the operation and follow-up were blinded to the allocated intervention according to the protocol.

### Statistical analysis

All the statistical analyses were performed using the SPSS software (version 20.0). The normal distribution of data was evaluated using Shapiro–Wilk (SW) test. Continuous variables were described as mean ± standard deviation (SD) or median (interquartile range, IQR) if data were not normally distributed. Continuous data were compared using a two-sample independent *t*-test or Mann–Whitney U test based on distribution. Categorical data were expressed as numbers and percentages and compared using chi-square for Fisher's exact test when appropriate. A two-sided *p*-value <0.05 was considered statistically significant.

## Results

**Figure 1:**
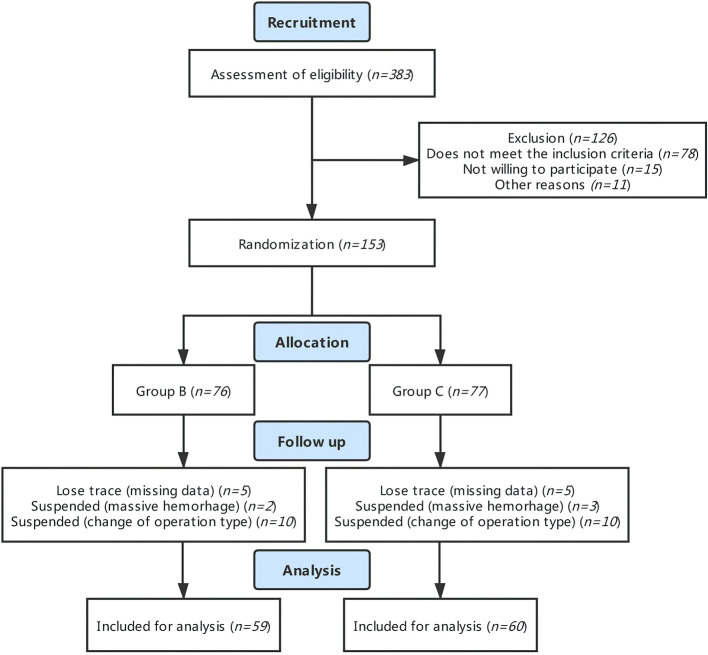
CONSORT flow diagram.

A total of 153 patients who met the eligibility criteria were enrolled and randomized in our study. Among them, 76 were assigned to the PTB group and 77 were assigned to the control group. However, during follow-up, 20 cases were excluded due to alterations in intraoperative surgical regimen, 5 cases were terminated in that intraoperative loss of blood was greater than 1,000 ml, and 9 cases were excluded from the analysis due to patients' withdrawal from the study and unavailability of blood sample. Therefore, 119 cases in total were analyzed in this study (59 cases in the PTB group and 60 cases in the control group) (Figure 1).

There were no statistically significant differences between the PTB group and the control group in the aspects of preoperative demographic and clinical characteristics (*p* > 0.05) (Table 1).

**Table 1:** Demographic and clinical characteristics of participants.

	PTB group (*n *= 59)	Control group (*n *= 60)	*p*-Value
Age, y	56.73 ± 8.89	55.65 ± 8.68	0.504
Gender, male, *n* (%)	36 (61.0)	28 (46.7)	0.142
BMI, kg/m^2^	23.14 ± 2.83	22.59 ± 2.93	0.300
HR beats per minute	77.15 ± 12.29	74.35 ± 11.95	0.210
Mean artery pressure, mmHg	93.80 ± 11.81	92.02 ± 13.27	0.442
Type of surgery			0.471
Pancreatoduodenectomy, *n* (%)	35 (59.3)	42 (70.0)	
Pancreatectomy, *n* (%)	21 (35.6)	16 (26.7)	
Partial Pancreatectomy, *n* (%)	3 (5.1)	2 (3.3)	
Operation time, min	162.45 ± 48.79	179.58 ± 69.70	0.125
Serum inflammatory markers			
IL-1β, pg/mg	5.00 (5.00,5.00)	5.00 (5.00,5.00)	0.818
IL-6, pg/mg	3.60 (2.29,5.45)	3.07 (2.00,5.37)	0.351
IL-8, pg/mg	27.90 (11.50,86.80)	20.30 (12.00,47.60)	0.341
IL-10, pg/mg	5.00 (5.00,5.00)	5.00 (5.00,5.00)	0.952
IL-2R, µ/ml	369.50 (296.25,436.25)	363.00 (296.00,501.00)	0.871
TNFα, pg/mg	8.96 (6.65,16.08)	9.53 (5.63,16.40)	0.985
CRP, mg/L	3.87 (1.82,6.61)	4.36 (2.05,8.85)	0.629
Percentile of Treg, %	17.35 (7.90,47.45)	20.00 (7.91,46.50)	0.603
Counts of Treg, *n*	11,181.07 (3409.76,28,328.64)	10,964.16 (2097.67,24,712.81)	0.894

Data were expressed as mean ± SD or median (IQR) or number (percentage). No statistically significant differences were found in baseline characteristics.

PTB group, bilateral paravertebral blockade group; Control group, control group; BMI, body mass index; HR, heart rate; IL, interleukin; TNFα, tumor necrosis factor alpha; CRP, C-reactive protein; Treg, regulatory T cells.

The perioperative consumption of opioids is displayed in Table 2. The mean dose of sufentanil perioperatively was 30.81 and 56.17 µg for the PTB group and the control group, respectively (*p* < 0.001). Considering the actual difference between the group, and that more patients than the minimum sample size required were recruited due to concern of dropout, the final analysis was with a calculated power of 99.9%. In addition, the intraoperative dose of sufentanil in the PTB group had a mean value of 9.58 µg, significantly lower than 33.67 µg in the control group (*p* < 0.001). The average postoperative dose of remifentanil was 1.01 mg in the PTB group, whereas 1.34 mg in the control group. There were statistically significant differences in the perioperative and intraoperative doses of sufentanil but not in the postoperative doses of sufentanil and remifentanil.

**Table 2:** Perioperative consumption of opioids.

	PTB group (*n *= 59)	Control group (*n *= 60)	*p*-Value
Perioperative dose of sufentanil, µg	30.81 ± 12.11	56.17 ± 35.73	0.000*
Intraoperative dose of sufentanil, µg	9.58 ± 12.12	33.67 ± 33.93	0.000*
Postoperative dose of sufentanil, µg	96.10 ± 16.64	93.25 ± 14.26	0.317
Postoperative dose of remifentanil, mg	1.01 ± 1.21	1.34 ± 1.22	0.143

PTB, bilateral paravertebral blockade group; Control group, control group.

* Indicates statistically significant, *p* < 0.05.

Table 3 shows the measures of secondary outcomes. The serum level of IL-10 in the experimental group was higher than that in the control group (*p* = 0.034); there were no significant differences in other inflammatory indicators between the two groups. No statistical differences were identified in terms of NRS at 12 or 24 h and SPONVIS assessed at 24 h after surgery. Infection, pancreatic fistula, emptying disorder, and postoperative hemorrhage were reported as complications, where infection was the most common with 11 cases (19%) in the PTB group and 7 cases (12%) in the control group. However, no significant differences were observed in complications between the two groups. When comparing the administration of vasoactive drugs, the PTB group had a higher mean dose of phenylephrine relative to the control group and the difference was statistically significant (*p* < 0.001). There were no statistically significant differences in the amount of blood loss, urinary output, colloid, and the total amount of fluid infusion. Noteworthy, patients receiving paravertebral block received more crystalloid infusion compared with the control group and the difference was significant (*p* = 0.016). Anal exhaust time and total length of stay showed no significant differences in the two groups.

**Table 3:** Measures of secondary outcomes: postoperative biomarkers, pain score, simplified postoperative nausea and vomiting impact scale, complications, administration of vasoactive drugs, intraoperative fluid volume, anal exhaust time, and total length of stay.

	PTB group (*n *= 59)	Control group (*n *= 60)	*p*-Value
Serum inflammatory markers			
IL-1β, pg/mg	5.00 (5.00,5.00)	5.00 (5.00,5.00)	0.432
IL-6, pg/mg	37.50 (22.80,66.60)	42.65 (21.20,67.73)	0.987
IL-8, pg/mg	68.60 (25.30,158.00)	61.25 (23.86,126.23)	0.520
IL-10, pg/mg	8.13 (5.55,12.30)	5.94 (5.00,9.87)	0.034
IL-2R, µ/ml	387.00 (313.00,456.00)	436.00 (324.00,570.00)	0.062
TNFα, pg/mg	8.09 (6.39,11.50)	7.83 (6.79,10.88)	0.857
CRP, mg/L	10.12 (6.06,18.00)	9.43 (7.02,16.66)	0.840
Percentile of Treg, %	20.80 (5.07,57.30)	19.05 (5.11,41.05)	0.405
Counts of Treg, *n*	2654.18 (501.77,8482.25)	1639.89 (485.79,8179.84)	0.663
NRS at 12 h	3.00 (2.00,3.00)	3.00 (3.00,3.00)	0.377
NRS at 24 h	3.00 (2.00,3.00)	3.00 (2.00,3.00)	0.676
SPONVIS	0.68 ± 1.20	0.80 ± 1.20	0.580
Complications			
Infection, *n* (%)	11 (19)	7 (12)	0.210
Pancreatic fistula, *n* (%)	3 (5)	2 (3)	0.492
Emptying disorder, *n* (%)	2 (3)	3 (5)	0.508
Postoperative hemorrhage, *n* (%)	1 (2)	1 (2)	0.748
Administration of vasoactive drugs			
Numbers of patients using ephedrine, *n* (%)	47 (79.7)	46 (76.7)	
Dose of ephedrine, mg	10.51 ± 9.08	9.02 ± 8.65	0.361
Numbers of patients using phenylephrine, *n* (%)	49 (83.1)	43 (71.7)	
Dose of phenylephrine, mg	0.61 ± 0.82	0.34 ± 0.65	0.000*
Intraoperative fluid volume			
Amount of blood loss, ml	466.78 ± 369.89	477.00 ± 407.94	0.886
Amount of urinary output, ml	456.78 ± 248.52	506.67 ± 279.75	0.306
Amount of fluid infusion, ml			
Crystalloid	1386.44 ± 593.49	1145.00 ± 477.43	0.016*
Colloid	1222.03 ± 570.56	1301.67 ± 717.69	0.505
Total	2608.47 ± 758.69	2446.67 ± 819.36	0.266
Anal exhaust time, d	3.10 ± 1.26	3.07 ± 1.13	0.873
Total length of stay, d	12.14 ± 6.52	11.45 ± 4.84	0.516

Data were expressed as mean ± SD or median (IQR) or number (percentage).

IL, interleukin; TNFα, tumor necrosis factor alpha; CRP, C-reactive protein; Treg, regulatory T cells; NRS, numerical rating scale; SPONVIS, simplified postoperative nausea and vomiting impact scale; NA, not available; PTB group, bilateral paravertebral blockade group; Control group, control group.

* Indicates statistically significant, *p* < 0.05.

## Discussion

To our knowledge, the present study was the first randomized trial investigating the efficacy of pain relief and safety with paravertebral block in resection surgery of pancreatic cancer. To be specific, we found that paravertebral block was significantly associated with reduced consumption of opioids perioperatively, as well as increased level of postoperative IL-10. No differences were found in postoperative complications, anal exhaust time, and total length of stay.

Consistent with previous studies (27–31), this study illustrated that bilateral paravertebral block combined with general anesthesia can reduce the perioperative consumption of opioids by approximately 45%, and reduce the intraoperative consumption of opioids by nearly 72%. Although it has been widely assumed that local anesthesia including paravertebral block should have alike effects, data from strict clinical studies were still lacking. Therefore, the results of this study confirmed that paravertebral block indeed could reduce opioid consumption as epidural block and may serve as an alternative in anaesthetization regimen. Postoperative dose of opioids showed no difference between the two groups since no postoperative continuous paravertebral block was administrated.

We also measured the serum level of inflammatory markers since they were suggested to be associated with postoperative stress, chronic pain as well as cancer progression. IL-10 was the only cytokine with a significant difference between the two groups. IL-10 is involved in versatile biological functions as a powerful immune mediator (32). The hypothesis that the presence of IL-10 during cancer would enhance the immune function of patients has been proposed (32, 33). Prior evidence suggested that various opioids have dose-related effects on immunosuppression (34). The results of this study gave the first hint that paravertebral block in open pancreatic surgery results in the increase of IL-10 may be due to reduced sufentanil that can function as an inhibitor of immune repression.

At 12 and 24 h after surgery, the analgesic effect of bilateral paravertebral block assessed by NRS showed no significant improvement. The same results were reported in several studies (28, 30). This may relate to the duration of the analgesic effect of paravertebral block. The median time of persistent analgesic effects for paravertebral block was 13 h (18). In this trial, the local anesthetic was injected through the catheter 1 h before the end of the operation. Hence, the analgesic effect of the paravertebral block might attenuate or disappear at 12 h after the operation.

This trial has certain limitations. First, the study mainly focused on immediate pain control and opioid usage during the perioperative period. More detailed analysis during the entire postoperative period would provide more information related to the chronic effects of opioid usage and be warranted in future studies. Also, the lack of control over when and why anesthetists or nurses gave opioids after surgery may cause bias in the results. Moreover, the variances in techniques for paravertebral block and manipulation methods during operation may produce inconstant results. For the biochemical measurement, postoperative change of cytokines is a dynamic process. The single timepoint assessment at 6 h may not represent sufficient results for inflammation evaluation.

In summary, in a rarely studied field of pancreatic resection, we showed that bilateral thoracic paravertebral block combined with general anesthesia reduced the consumption of opioids in the perioperative period by 45% and may decrease inhibition of opioids on serum concentration of IL-10, a key anti-inflammatory mediator. Yet, cautions should be taken of potential loss of intravascular volume and hypotension.

## Contribution to the field statement

Opioids including fentanyl and its derivatives and morphine are considered as the pillar stone of pain management in the surgical process. However, opioid usage has been reported to be correlated with certain adverse outcomes after the pancreatic section. Thus, reduction of opioids, a common goal in surgical analgesia strategy, may be of great importance in pancreatic resections of cancer patients. Our study found that paravertebral block reduced the opioid requirement during the perioperative period, which may be through the effect of an inflammatory mediator such as IL-10. Given the fact that the majority of anesthesiologists still have a preference for epidural blockade or other alternative analgesic regimens and limited clinical studies have been conducted with paravertebral block, especially in pancreatic cancer surgery, our study can provide further evidence for the benefits of the application of this technique.

## Data Availability

The raw data supporting the conclusions of this article will be made available by the authors, without undue reservation.
